# Metastases to inguinal lymph nodes in prostate cancer: a new perspective on an uncommon pattern of spread

**DOI:** 10.1590/0100-3984.2024.57.e4-en

**Published:** 2024-08-02

**Authors:** Alice Schuch

**Affiliations:** 1 Abdominal Radiologist, Head of the Department of Radiology and Diagnostic Imaging at the Hospital Moinhos de Vento, Porto Alegre, RS, Brazil. Email: alice.schuch@hmv.org.br; aliceschuch@gmail.com

Prostate cancer is the second most common malignancy in men, second only to non-melanoma skin cancer^(1)^. The metastatic spread of prostate cancer occurs through local in-vasion, lymphatic dissemination, and hematogenous dissemi-nation, the most common sites being the pelvic lymph nodes and bones, where metastases are found in 99% and 84% of cases, respectively^(2)^. The question of inguinal lymph node in-volvement has emerged primarily due to the increased use of cross-sectional imaging methods such as CT and MRI, as well as metabolic evaluation by PET, especially with prostate-spe-cifc radiotracers such as PSMA. Metastases to inguinal lymph nodes are considered rare and present a diagnostic challenge because they are not typically included in conventional treat-ment felds such as radiotherapy or lymphadenectomy. The actual prevalence of and predictive factors for metastatic inguinal lymph nodes remain uncertain, and data in the litera-ture are based on imaging fndings without histopathological correlation^(3,4)^.

The study “Inguinal lymph node metastases from prostate cancer: clinical, pathology, and multimodality imaging con-siderations”^(5)^, recently published in Radiologia Brasileira, comprehensively investigates the clinical, pathological, and imaging fndings associated with metastases in inguinal lymph nodes in patients with prostate cancer who underwent image-guided inguinal lymph node biopsy. Through a detailed retro-spective analysis involving a substantial cohort of patients, the authors explored morphological and functional characteristics, such as inguinal lymph node short-axis diameter, PSMA uptake on PET, and extent of the primary tumor, as well as complete staging, with assessment of the pattern of lymphatic spread and non-lymph node metastases. Clinical, laboratory, and pre-vious treatment data (primary tumor or recurrence) were also crucial in the assessment of metastatic inguinal lymph nodes, including PSA levels and castration resistance.

This study contributes signifcantly to the understanding of risk factors for inguinal lymph node metastases in prostate cancer, being the largest cohort for this assessment. Knowl-edge of these risk factors can help radiologists and nuclear medicine physicians interpret fndings, which are essential for accurate patient staging and individualized therapeutic plan-ning. The authors emphasize that isolated inguinal lymph node metastasis is extremely rare in the absence of identifed risk factors. Additionally, investigation with different diagnostic methods, including PSMA-PET and potentially PET with radio-tracers other than PSMA, in the detection of inguinal lymph nodes metastases, is promising for a more accurate diagnosis. As a future perspective, the investigation of biomarkers and molecular pathways involved in the spread of prostate cancer to inguinal lymph nodes may pave the way for therapies that are more targeted and personalized^(6)^.
